# Suspected allergic bronchopulmonary candidiasis in a patient with concurrent multidrug-resistant bacterial pneumonia: A case report

**DOI:** 10.1097/MD.0000000000050977

**Published:** 2026-10-02

**Authors:** Xianxian Wu, Tongxin Zang, Yingbei Liu, Bo Xu

**Affiliations:** a The Second Department of Neurology, Qingdao Central Hospital, University of Health and Rehabilitation Sciences (Qingdao Central Hospital), Qingdao, Shandong, China; b Emergency Trauma Surgery, Qingdao Central Hospital, University of Health and Rehabilitation Sciences (Qingdao Central Hospital), Qingdao, Shandong, China; c The First Department of Neurology, Qingdao Central Hospital, University of Health and Rehabilitation Sciences (Qingdao Central Hospital), Qingdao, Shandong, China.

**Keywords:** allergic bronchopulmonary candidiasis, allergic bronchopulmonary mycosis, *Candida albicans*, next-generation sequencing

## Abstract

**Rationale:**

Allergic bronchopulmonary candidiasis (ABPC) is a rare pulmonary hypersensitivity disorder; diagnosis is difficult because *Candida albicans* is usually a respiratory commensal.

**Patient concerns:**

A 70-year-old man with hypertension and prior cerebral infarction was admitted for sudden right limb weakness (6.5 hours) and diagnosed with hemorrhagic cerebral infarction in the intensive care unit.

**Diagnoses:**

Persistent fever and pulmonary infiltrates developed despite broad-spectrum antibiotics. Total immunoglobulin E (IgE) was 2181 IU/mL; sputum grew *C albicans* + carbapenem-resistant *Pseudomonas aeruginosa*; bronchoalveolar lavage fluid metagenomic next-generation sequencing detected *C albicans*, *P aeruginosa*, *Klebsiella pneumoniae*, EBV, and HSV-1. ABPC was suspected alongside multidrug-resistant bacterial pneumonia.

**Interventions:**

Ceftazidime-avibactam (2.5 g q8h) + nebulized polymyxin B (0.5 M IU q12h); oral voriconazole (400 mg q12h loading, then 200 mg q12h); IV methylprednisolone 40 mg/d.

**Outcomes:**

Clinical improvement on combined therapy; respiratory stabilization within 2 weeks; intensive care unit transfer out. Follow-up chest computed tomography showed resolution of infiltrates; total IgE fell from 2181 to 995 IU/mL (–54%). Discharged with hemorrhagic cerebral infarction, hypertension, carbapenem-resistant *Pseudomonas pneumonia*, *K pneumoniae* co-infection, and ABPM.

**Lessons:**

Persistent infiltrates with high IgE and Candida isolation should suggest ABPM, not intensified antibiotics. ABPC can occur in older patients without asthma. Early corticosteroids plus antifungals yield rapid improvement. Metagenomic next-generation sequencing complements conventional testing but cannot confirm ABPC alone.

## 1. Introduction

Allergic bronchopulmonary mycosis (ABPM) is a lung disorder identified as hypersensitivity immune responses to inhaled fungals.^[1]^ The most common type of ABPM is allergic bronchopulmonary aspergillosis (ABPA), caused by *Aspergillus fumigatus*. ABPA most often occurs in patients with conditions including asthma, cystic fibrosis, chronic obstructive pulmonary disease, or bronchiectasis.^[2]^

Allergic bronchopulmonary mycosis caused by fungi other than *Aspergillus*, such as *Candida albicans* – known as allergic bronchopulmonary candidiasis (ABPC) – is uncommon.^[3]^ Diagnosing ABPC is difficult because *C albicans* is often regarded as a commensal of the airways rather than invasive. In 2024, the International Society for Human and Animal Mycology (ISHAM) revised the diagnostic standards for ABPM. According to these criteria, a diagnosis requires a preexisting condition like asthma or cystic fibrosis, an elevated total serum immunoglobulin E (IgE) level (≥500 IU/mL), and fungal sensitization demonstrated by a positive skin test or the presence of fungus-specific IgE.^[4]^ For ABPM caused by non-*Aspergillus* fungi, the guideline further recommended that the fungus should be isolated from sputum or detected in bronchoalveolar lavage fluid (BALF).^[5]^ Treatment is based on oral corticosteroids combined with antifungals to control the immune response and reduce the fungal burden in the airways.^[4]^

Herein, we report on a severe manifestation of ABPC in a critically ill patient with a hemorrhagic stroke, in whom the diagnosis was established with the aid of metagenomic next-generation sequencing (mNGS).

## 2. Case presentation

The timeline of this case is shown in Figure 1. On July 2, 2025, a 70-year-old retired man was admitted to the emergency department due to sudden onset of right limb weakness lasting for 6.5 hours. He was diagnosed with hemorrhagic cerebral infarction and admitted to the intensive care unit (ICU) for further management. The patient had a history of hypertension (2 years), old cerebral infarction with mild cognitive impairment, and pulmonary nodules of unclear pathology. He had smoked half a pack per day for more than 40 years but denied alcohol abuse. No history of diabetes, coronary artery disease, tuberculosis, or drug allergies was reported.

**Figure 1.**
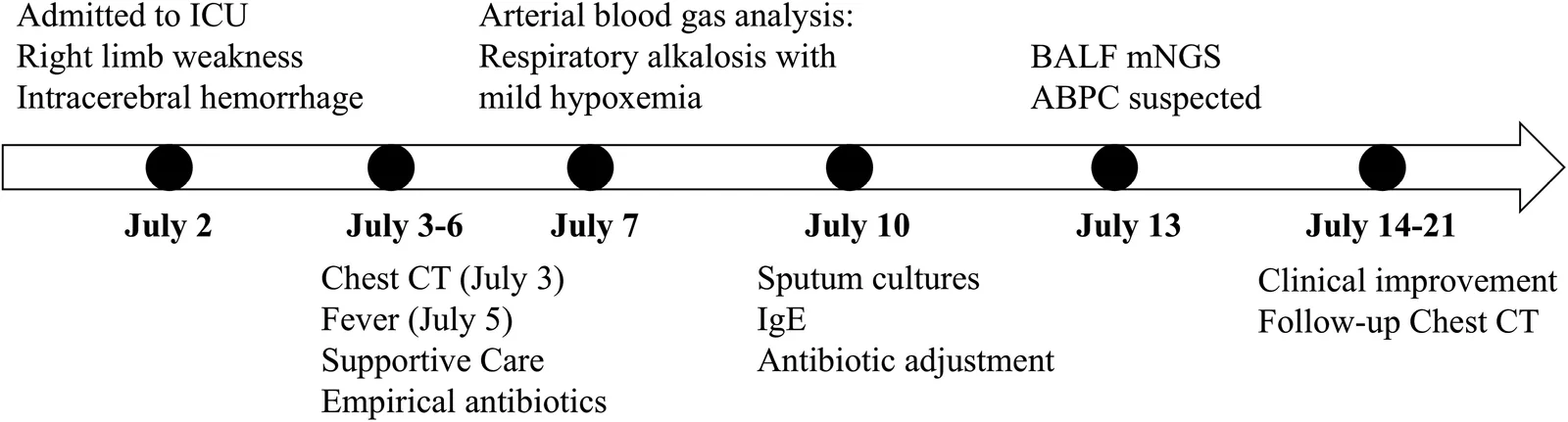
Timeline of the case. ABPC = allergic bronchopulmonary candidiasis, BALF = bronchoalveolar lavage fluid, CT = computed tomography, ICU = intensive care unit, IgE = immunoglobulin E.

On admission, the patient was somnolent but arousable. Physical examination revealed right-sided hemiparesis (muscle strength 4/5), slurred speech, and Babinski sign on the right side. Pulmonary auscultation revealed coarse breath sounds without rales. Chest computed tomography (CT) revealed diffuse interstitial changes with bronchial wall thickening, bronchiectasis, and mucus impaction (Fig. 2A). Routine laboratory tests showed leukocytosis (white blood cell [count] 13.1 × 10^9^/L, neutrophils 12.14 × 10^9^/L) and mildly elevated C-reactive protein (8.72 mg/L), as summarized in Table 1. The patient received supportive care for intracerebral hemorrhage infarction, including blood pressure control, dehydration therapy, and empirical antibiotics (piperacillin-tazobactam) for suspected aspiration pneumonia.

**Table 1:** Laboratory results: CRP, total IgE, WBC, ALT, and creatinine.

Date	07-03	07-05	07-06	07-08	07-10	07-11	07-14	07-16	07-22
CRP (mg/L)	8.720	15.260	30.820	82.640		66.540	12.420	5.000	20.640
Total IgE (IU/mL)			2181		1529		995		
WBC (×10^9^/L)	13.12	9.54	8.86	10.81		12.65	11.55	14.04	11.07
ALT (U/L)	17	13	25	54		33	30	30	
Creatinine (µmol/L)	72	77	73	65		51	57	59	

ALT = alanine aminotransferase, CRP = C-reactive protein, IgE = immunoglobulin E, WBC = white blood cell count.

**Figure 2.**
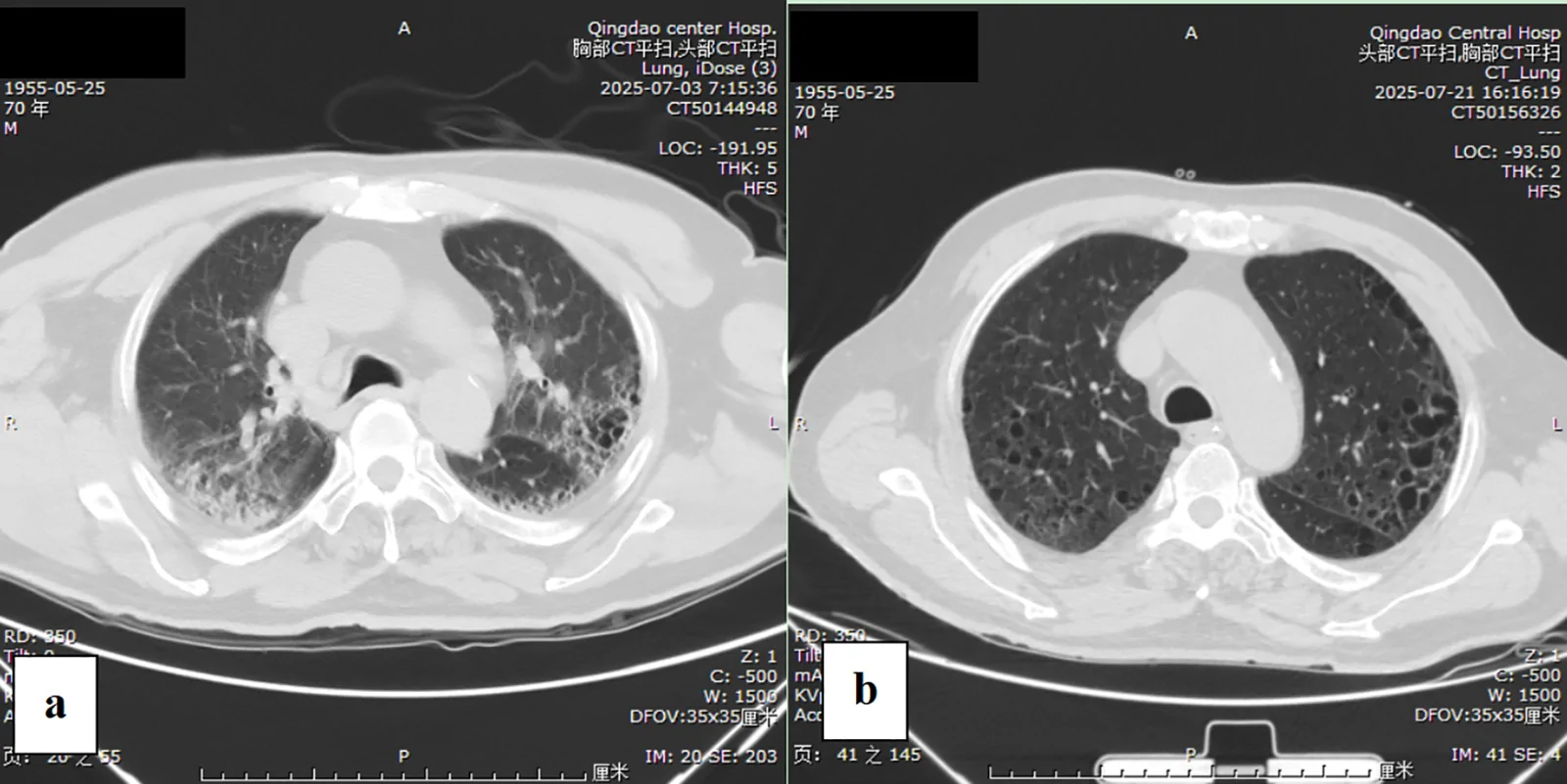
Chest CT of the patient. (A) On July 3, 2025, chest CT showed diffuse interstitial infiltrates with bronchial wall thickening in both lungs. (B) On July 21, 2025, chest CT showed resolution of pulmonary infiltrates after corticosteroid and antifungal therapy. CT = computed tomography.

During hospitalization, the patient developed recurrent fever (up to 38.5°C) from July 5 despite broad-spectrum antibiotic therapy. Arterial blood gas analysis (July 7, 2025) showed alkalosis (pH 7.48), decreased partial pressure of carbon dioxide (PaCO_2_ 27 mm Hg), and mildly reduced partial pressure of oxygen (PaO_2_ 75 mm Hg), with a normal lactate level (1 mmol/L). The findings indicated respiratory alkalosis with mild hypoxemia. The patient had no apparent dyspnea and was managed with mask oxygen supplementation. On July 10, sputum cultures showed growth of *C albicans* and carbapenem-resistant *Pseudomonas aeruginosa*. Allergen testing revealed a markedly elevated total IgE level of 2181 IU/mL. The consultation recommended adjustment of antimicrobial therapy according to susceptibility results: ceftazidime–avibactam (2.5 g every 8 hours, prolonged infusion) combined with nebulized polymyxin B (0.5 million IU every 12 hours), and initiation of methylprednisolone (40 mg/d, intravenous) and oral voriconazole (400 mg every 12 hours on day 1, then 200 mg every 12 hours). On July 13, mNGS of bronchoalveolar lavage fluid (BALF) revealed the presence of *P aeruginosa*, *Klebsiella pneumoniae*, *C albicans*, Epstein–Barr virus, and human herpesvirus type 1. Because the respiratory manifestations developed more than 48 hours after ICU admission and multidrug-resistant *P aeruginosa* was identified, concurrent hospital-acquired bacterial pneumonia was also considered. Based on the high total IgE levels, radiologic features, clinical course, and mNGS results, allergic bronchopulmonary candidiasis (ABPC) was suspected. The patient’s clinical condition improved after combined antibacterial, antifungal, and corticosteroid therapy; however, the response could not be attributed to any single component of the regimen. Inflammatory markers were scheduled for reevaluation the following day to guide antibiotic de-escalation.

After approximately 2 weeks of corticosteroid and antifungal therapy, the patient’s respiratory status stabilized, and inflammatory markers decreased. He was transferred from the ICU to the neurology ward for continued rehabilitation of stroke-related deficits. A follow-up chest CT performed on July 21 showed significant improvement in pulmonary infiltrates (Fig. 2B). At discharge, diagnoses included hemorrhagic cerebral infarction, hypertension (grade 2, very high risk), carbapenem-resistant *Pseudomonas pneumonia*, *K pneumoniae* co-infection, and allergic bronchopulmonary mycosis caused by *C albicans*.

## 3. Discussion and conclusion

Various fungal infections can occur in the lungs, and each may lead to allergic sensitization. Therefore, fungal allergens should be considered in patients with pulmonary infiltrates unresponsive to antibiotic therapy.^[6-9]^

Allergic bronchopulmonary mycosis (ABPM) is defined as a hypersensitivity-mediated airway disorder resulting from type I and III immune reactions to fungal antigens inhaled and colonized in the bronchi, leading to bronchial inflammation, mucus impaction, and bronchiectasis. The prototypical form, allergic bronchopulmonary aspergillosis (ABPA), is most commonly caused by *A fumigatus* in patients with asthma or cystic fibrosis.^[5]^ Allergic bronchopulmonary candidiasis (ABPC) was first reported in the early 1980s,^[10,11]^ but remains under-recognized. Over the past decade, sporadic ABPC cases have been documented, with clinical severity ranging from mild bronchial inflammation to respiratory failure.^[3,12,13]^

While ABPA dominates the ABPM spectrum, non-Aspergillus fungi such as *C albicans*, Bipolaris, or Schizophyllum commune can also provoke similar hypersensitivity responses. These entities are often misdiagnosed because *Candida* is regarded as a commensal organism rather than a pathogen. Epidemiologic analyses suggest that 47% of global ABPM cases unrelated to Aspergillus occur in India; most of them are caused by *C albicans.*^[3]^

The diagnosis of ABPM requires integration of clinical, immunological, radiological, and microbiological findings. Our patient had compatible radiological findings, a markedly elevated total serum IgE level, and detection of *C albicans* in sputum and BALF. However, *Candida*-specific IgE or skin testing was not performed, and repeated fungal isolation was not documented. Therefore, the current diagnostic criteria for ABPM were not fully satisfied, and ABPC should be regarded as a suspected rather than definitively confirmed diagnosis.

Total IgE can be elevated in various allergic diseases; however, an IgE level higher than 1000 IU/mL is typical for ABPM.^[14]^ Some investigators have proposed even higher cutoff values to improve specificity in diagnosing ABPM.^[15]^ Monitoring total IgE during therapy is advised because in some cohorts, a decline of 35 % or greater has been associated with a positive treatment response.^[16,17]^ Moreover, ISHAM guidelines suggest a 20 % reduction may indicate improvement.^[4]^

The demonstration of *Candida*-specific IgE confirms a type I hypersensitivity mechanism, though it may sometimes reflect environmental exposure.^[7]^ Similarly, the recovery of *C albicans* in sputum is supportive but not diagnostic, as colonization is common in patients using inhaled corticosteroids.^[18,19]^ Hence, interpretation requires integration of microbiologic, serologic, and radiologic data.

Peripheral blood eosinophilia, traditionally a minor criterion, may be absent due to corticosteroid suppression. Some ABPM patients exhibit normal eosinophil counts.^[2,20]^ Thus, a normal eosinophil level should not exclude the diagnosis if other parameters are fulfilled.

Serum galactomannan (GM) and (1,3)-β-D-glucan (BDG) are reliable for invasive fungal infections but are typically within normal limits in ABPM.^[21]^ This pattern supports an allergic, noninvasive process. In our patient, both serum and BALF GM/BDG were negative, effectively ruling out invasive disease.

Radiographically, ABPM usually presents as transient pulmonary opacities (“fleeting shadows”). High-resolution CT (HRCT) of the chest in ABPM/ABPA commonly demonstrates central (proximal) bronchiectasis and mucoid impaction; in a subset (20–40%) of cases, the mucus appears hyper-attenuating (high-attenuation mucus), a finding strongly associated with disease severity.^[22-24]^ In our case, the CT scan demonstrated bronchial wall thickening and mucus impaction, consistent with allergic fungal involvement.

ABPC shares the same pathophysiological basis as ABPA, involving type I (IgE-mediated) and type III (immune-complex) hypersensitivity reactions. The interaction of fungal antigens with bronchial mucosa triggers T-helper type 2 (Th2) cytokine release, IgE production, and eosinophil recruitment, leading to airway obstruction and remodeling.^[25]^

To note, this case is absent of preexisting conditions including asthma or cystic fibrosis, suggesting that *Candida* can still cause disease even in the healthy lung.

According to guidelines, treatment aims to suppress the hypersensitivity response with corticosteroids and decrease fungal burden with antifungal drugs. Systemic corticosteroids are first-line therapy, as the regimens recommend prednisone 0.5 mg/kg/d for 2 to 4 weeks with gradual tapering.^[12]^ Clinical symptom improvement usually parallels a decline in serum IgE and resolution of CT findings.

The role of antifungal drugs in treating ABPM remains debated. Agents such as fluconazole, itraconazole, and voriconazole can help lower fungal antigen burden but may cause liver toxicity and promote resistance.^[26,27]^ In this case, combined use of corticosteroids and antifungal drugs led to fast recovery, aligning with previous reports.^[3,12,28]^

Current data on inhaled corticosteroids or nebulized antifungal agents are insufficient, and these should not replace systemic treatment. Novel biologic options, such as anti-IgE therapy, have shown promise in refractory ABPA and may be useful in steroid-dependent ABPM.^[29,30]^

This case provides several practical insights. Persistent lung infiltrates with high IgE and *Candida* isolation in sputum should raise suspicion for ABPM rather than introducing additional antibiotic treatment. ABPC can develop even in older patients without asthma but with multiple comorbidities. Early corticosteroid therapy with antifungal drugs can produce fast improvement and help avoid permanent lung injury.

This case has several limitations. First, the absence of *Candida*-specific sensitization testing and repeated fungal culture prevented definitive confirmation of ABPC. Second, concurrent multidrug-resistant bacterial pneumonia and the simultaneous administration of antibacterial, antifungal, and corticosteroid therapy made it impossible to determine which condition or treatment accounted for the clinical and radiological improvement. Finally, mNGS detection alone could not distinguish airway colonization from clinically relevant fungal involvement.

Regular follow-up is needed to monitor serum IgE levels, radiological changes, and possible disease recurrence. In conclusion, ABPC may be considered in patients with compatible radiological findings, markedly elevated total IgE, and microbiological evidence of *Candida* involvement. However, mNGS detection alone does not establish causality, particularly in critically ill patients with concurrent bacterial pneumonia. Diagnostic findings and treatment responses should therefore be interpreted cautiously and in combination with fungal sensitization testing, conventional microbiological examinations, imaging findings, and clinical follow-up.

## Author contributions

**Conceptualization:** Xianxian Wu.

**Data curation:** Xianxian Wu, Yingbei Liu.

**Formal analysis:** Tongxin Zang.

**Investigation:** Tongxin Zang.

**Methodology:** Tongxin Zang.

**Project administration:** Bo Xu.

**Resources:** Yingbei Liu.

**Supervision:** Bo Xu.

**Validation:** Yingbei Liu.

**Writing – original draft:** Xianxian Wu.

**Writing – review & editing:** Xianxian Wu, Tongxin Zang, Bo Xu.
